# A recombinant Marek’s disease vaccine candidate provides complete protection against infectious bursal disease virus and H9 subtype avian influenza virus in chickens

**DOI:** 10.1128/jvi.01149-25

**Published:** 2025-09-11

**Authors:** Wenrui Fan, Xianying Zeng, Yuntong Chen, Qingqing Yu, Zibo Zhang, Guobin Tian, Changjun Liu, Hongmei Bao, Xiaole Qi, Longbo Wu, Yanping Zhang, Yongzhen Liu, Suyan Wang, Hongyu Cui, Yulu Duan, Hualan Chen, Yulong Gao

**Affiliations:** 1 State Key Laboratory for Animal Disease Control and Prevention, The Chinese Academy of Agricultural Sciences, Harbin Veterinary Research Institute 111613 , Harbin, PR China; Icahn School of Medicine at Mount Sinai, New York, New York, USA

**Keywords:** MDV-1, IBDV, H9 subtype AIV, VP2, HA, vaccine

## Abstract

Vaccination is the most effective preventative measure against economically devastating poultry diseases, such as infectious bursal disease (IBD), H9 subtype avian influenza (AI), and Marek’s disease. In recent decades, various vaccination strategies have been investigated and developed. Among these, multivalent and combination vaccines, which confer protection against multiple diseases in a single dose, have emerged as a significant advancement in veterinary medicine. This study demonstrates that the attenuated Marek’s disease virus serotype 1 (MDV-1) vaccine strain, rMSΔMeq, known for its safety and robust carrier immunogenicity, can serve as a vector for the heterologous expression of multiple antigenic proteins. We constructed the recombinant virus rMDV-VP2-HA by inserting the H9 subtype avian influenza virus (AIV) hemagglutinin (HA) expression cassette into the UL41 region and the infectious bursal disease virus (IBDV) VP2 expression cassette into the US2 region. The growth characteristics of this recombinant virus were consistent with those of the parental virus, and it stably expressed the HA and VP2 genes. Vaccination with rMDV-VP2-HA induces high titers of IBDV-neutralizing antibodies and hemagglutinin inhibition (HI) antibodies and elicits strong cellular immune responses. Specifically, vaccination with rMDV-VP2-HA enhances interferon-gamma (IFN-γ) expression in response to stimulation with HA and VP2 proteins. Meanwhile, cytokines associated with both Th1 and Th2 responses were also upregulated. Remarkably, chickens vaccinated with rMDV-VP2-HA achieved complete protection against very virulent IBDV, H9 subtype AIV, and very virulent MDV. These findings underscore the exceptional practical potential of rMDV-VP2-HA as the first MDV-1 vector-based multivalent vaccine candidate for combating these poultry diseases.

**IMPORTANCE:**

Commercial vaccines for infectious bursal disease (IBD) and H9 subtype avian influenza (AI) require multiple doses, increasing costs and causing stress in chicken flocks. Additionally, their efficacy is frequently compromised by maternal antibody interference. This underscores the urgent need for a multivalent, multi-component, and single-dose vaccines capable of streamlining immunization protocols, overcoming maternal antibody interference, and providing lifelong immunity. Our research demonstrates, for the first time, serotype 1 Marek’s disease virus (MDV-1) can stably express multiple exogenous genes. More significantly, the rMDV-VP2-HA elicits robust humoral and cellular immune responses, achieving complete protection against H9 subtype AIV, very virulent IBDV, and very virulent MDV with a single immunization. These findings contribute to enhancing the efficiency of disease prevention and confirm that the MDV-1 is an ideal vector for developing multivalent vaccines, achieving the goal of “multiple protections with a single shot.” This advancement represents a significant progression in the prevention and control of economically important poultry diseases.

## INTRODUCTION

Infectious bursal disease virus (IBDV) and H9 subtype avian influenza virus (AIV) are major pathogens that threaten both the poultry industry and public health (1–4). IBDV, a member of the *Birnaviridae* family and *Avibirnavirus* genus, causes acute and highly transmissible viral infection in chickens, primarily affecting the bursa of Fabricius. Very virulent strains of IBDV (vvIBDV) are particularly devastating, causing severe damage to the bursa and depletion of B lymphocytes, with mortality rates exceeding 60% (5, 6). This pathogen poses a significant threat to the sustainable development of the global poultry industry. The capsid of IBDV is strictly ordered and composed exclusively of VP2, the major protective antigen containing epitopes that induce neutralizing antibodies essential for the immune response (7).

The H9 subtype AIV, a predominant low-pathogenic influenza virus, also poses a significant economic burden on the poultry industry (8, 9). This virus often co-infects with other pathogens, exacerbating clinical disease severity and potentially leading to mortality (10, 11). Moreover, H9 subtype AIV serves as a gene donor for other AIV subtypes, including H5N1, H5N6, H7N9, and H10N8, which are zoonotic and pose a serious threat to human health (12–15). The envelope glycoproteins of AIV, particularly hemagglutinin (HA) protein, are the primary targets of the host’s immune response (16, 17).

Vaccination is an effective strategy to control the spread of these pathogens, with vector-based vaccines emerging as a promising approach for simultaneously preventing and managing multiple infections. Marek’s disease virus (MDV) is the most widely used vector for expressing protective antigens against avian pathogens. MDVs are categorized into three distinct serotypes: highly virulent serotype 1 (MDV-1), less pathogenic serotype 2 (MDV-2), and nonpathogenic serotype 3, also recognized as the Herpesvirus of Turkey (HVT) (18). Despite the widespread use of HVT as a vector, it has been reported that HVT alone is insufficient to protect against very virulent MDV (vvMDV) (19). In contrast, attenuated MDV-1 strains offer robust protection against these highly pathogenic variants, thereby showcasing a superior potential as a vector due to their enhanced immunogenicity and protective efficacy.

To achieve dual protection against IBD and H9 subtype AIV and simultaneously provide a comprehensive defense against Marek’s disease (MD) through a single viral vector, we constructed a recombinant MDV using the MDV-1 Meq gene-deleted MDV-1 vaccine strain (rMSΔMeq) as the vector (20). This recombinant virus, designated rMDV-VP2-HA, was engineered to co-express the HA gene from H9 subtype AIV and the VP2 gene from IBDV. Furthermore, we demonstrate that vaccination with rMDV-VP2-HA induces robust humoral and cellular immune responses in specific-pathogen-free (SPF) chickens. These immune responses are marked by high titers of IBDV-neutralizing and hemagglutination inhibition (HI) antibodies, along with significant upregulation of cytokines such as interferon-gamma (IFN-γ) and tumor necrosis factor-alpha (TNF-α). Furthermore, rMDV-VP2-HA vaccination provided complete protection against challenges from H9 subtype AIV, vvIBDV, and vvMDV in SPF chicken.

## RESULTS

### Successful rescue of recombinant MDVs containing both the VP2 and HA genes

**Fig 1:**
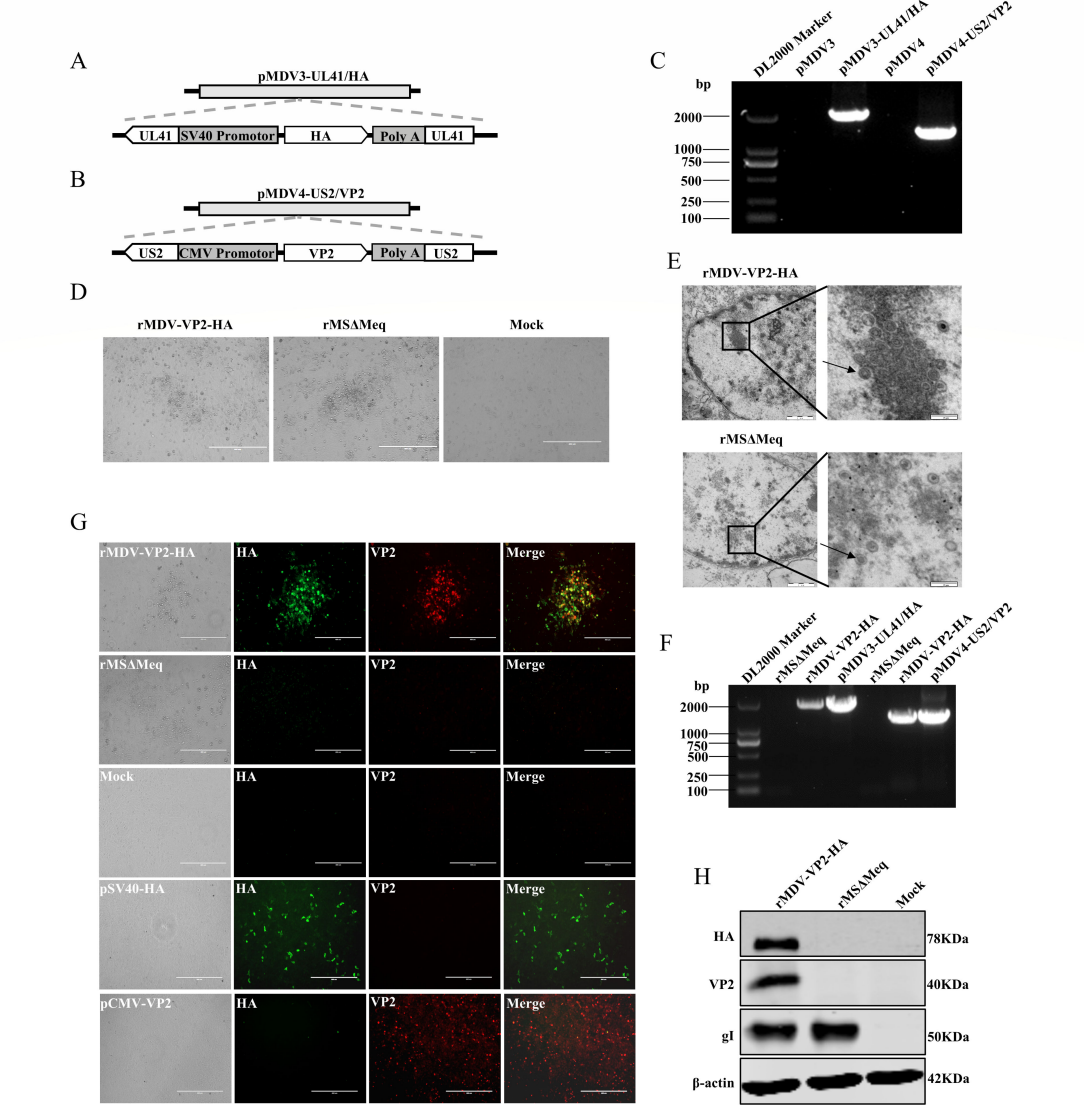
The recombinant MDVs containing both the VP2 and HA genes were successfully rescued. (**A**) Schematic diagrams of the fosmid containing the HA-expressing cassette. (**B**) Schematic diagrams of the fosmid containing the VP2-expressing cassette. (**C**) PCR amplification of the HA and VP2 gene from the recombinant fosmid pMDV3-UL41/HA and pMDV4-US2/VP2. (**D**) Cytopathic effects induced by a recombinant virus (rMDV-VP2-HA) and parental virus (rMSΔmeq) were observed via inverted microscopy (bar length, 400 µm). (**E**) Electron microscopy detection of recombinant virus (rMDV-VP2-HA) and parental virus (rMSΔmeq) in infected CEFs (bar length, 1 µm). (**F**) PCR amplification of the HA and VP2 gene from the rescued recombinant virus (rMDV-VP2-HA). (**G**) Detection of HA and VP2 from the recombinant virus in CEF with IFA, pSV40-HA, and pCMV-VP2 were used as positive control. (**H**) Detection of HA and VP2 expression in recombinant virus-infected CEF by Western blot. Glycoprotein I (gI) is a structural protein specific to MDV and was used as the internal reference protein for MDV in this experiment.

To construct the recombinant MDV, a series of fosmids (pMDV1, pMDV2, pMDV3, pMDV4, and pMDV5) encompassing the genome of the MDV-1 vaccine strain (rMSΔMeq) were employed as previously described (21). The HA gene was incorporated into the UL41 region of pMDV3, and the VP2 gene was incorporated into the US2 region of pMDV4, generating the recombinant fosmids pMDV3-UL41/HA and pMDV4-US2/VP2, respectively (Fig. 1A and B). Both of these recombinant fosmids were verified by polymerase chain reaction (PCR) analysis (Fig. 1C). To generate the recombinant virus, the recombinant plasmids pMDV3-UL41/HA and pMDV4-US2/VP2 were co-transfected with parental plasmids (pMDV1, pMDV2, and pMDV5) into chicken embryo fibroblast (CEF) cells. Following two serial passages in CEF, MDV-1-typical plaques were observed (Fig. 1D). Electron microscopy analysis further validated the successful rescue of the recombinant virus, which was similar to the parental virus (rMSΔMeq) (Fig. 1E). Precise integration of the VP2 and HA genes at their designated regions was confirmed by PCR amplification (Fig. 1F). Collectively, these findings demonstrate the successful rescue of the recombinant virus constructed from the rMSΔMeq vaccine strain with the insertion of the HA and VP2 genes, designated as rMDV-VP2-HA.

### rMDV-VP2-HA successfully expressed VP2 and HA gene

To confirm the expression of the inserted HA and VP2 genes in the rMDV-VP2-HA, immunofluorescence assay (IFA) and Western blot analyses were performed. In the IFA, HA expression (green) was detected using anti-HA polyclonal antibodies, whereas VP2 expression (red) was identified with anti-VP2 monoclonal antibodies (Fig. 1G). In the Western blot, rMDV-VP2-HA-infected cells showed positive reactivity with HA polyclonal antibodies and VP2 monoclonal antibodies, revealing an HA-specific band at approximately 78 kDa and a VP2-specific band at approximately 40 kDa (Fig. 1H). These results indicate that the rMDV-VP2-HA successfully expressed both HA and VP2 proteins.

### rMDV-VP2-HA demonstrates robust genetic stability

**Fig 2:**
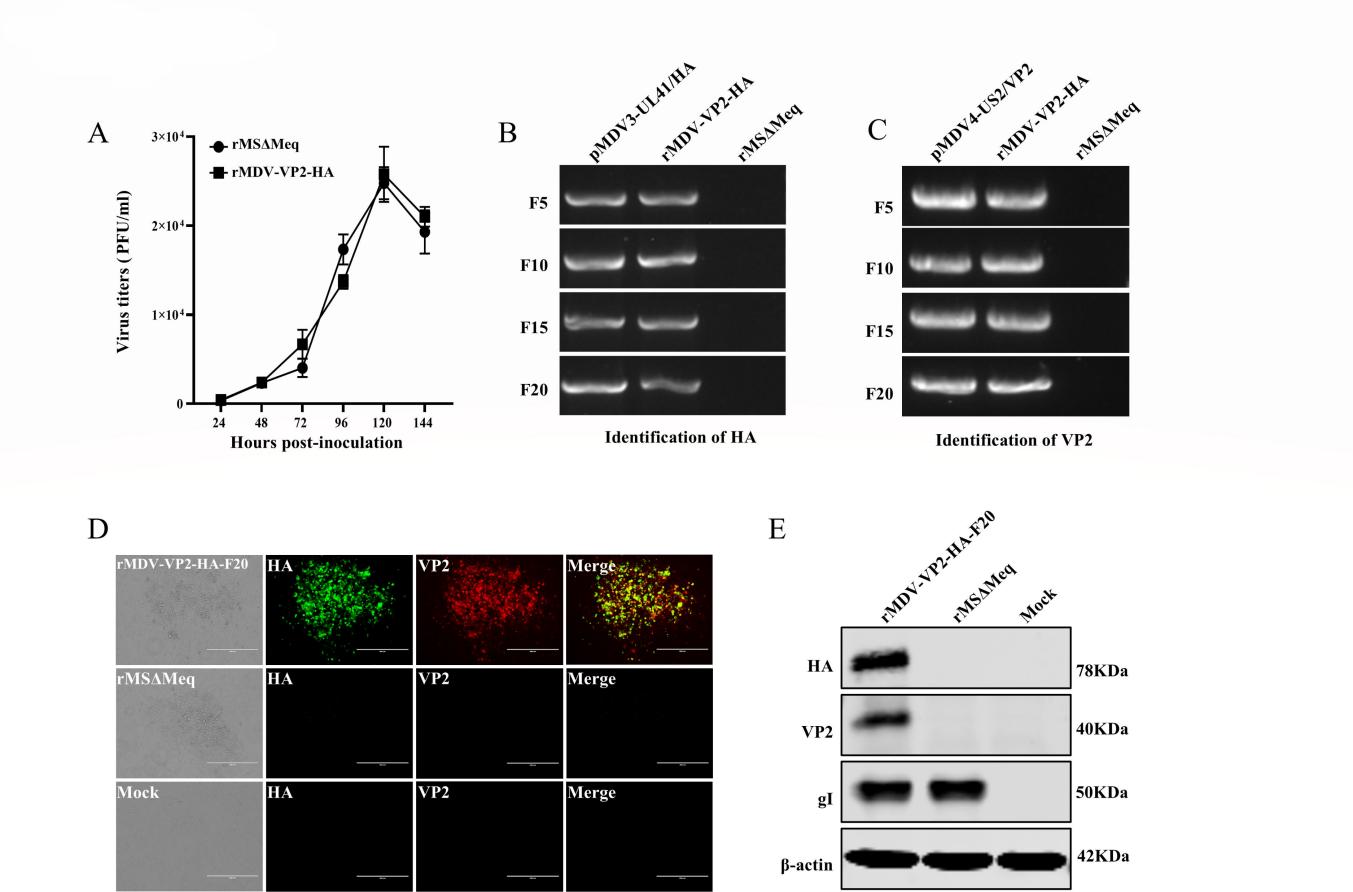
The rMDV-VP2-HA possesses good growth characteristics and genetic stability. (**A**) Growth kinetics of recombinant virus (rMDV-VP2-HA) and parental virus (rMSΔmeq). rMDV-VP2-HA and rMSΔmeq were inoculated into CEFs with 100 PFU per well. Cells were harvested and titered at 24, 48, 72, 96, 120, and 144 h post-inoculation. Error bars indicate standard deviations. (**B**) rMDV-VP2-HA was grown sequentially in CEF cells for 20 passages. The HA gene was examined every five passages by PCR amplification. (**C**) rMDV-VP2-HA was grown sequentially in CEF cells for 20 passages. VP2 genes were examined every five passages by PCR amplification. (**D**) Detection of the HA and VP2 from the recombinant virus was passaged 20 times in CEFs using IFA. (**E**) Detection of HA and VP2 from the recombinant virus was passaged 20 times in CEFs using Western blot.

To evaluate whether the insertion of the HA and VP2 genes affects the replication characteristics of rMDV-VP2-HA, its replication kinetics were analyzed. The results indicated that rMDV-VP2-HA achieved peak replication titers of 2.58 × 10^4^ PFU/ml at 120 hours post-infection, comparable with the parental virus (rMSΔMeq) (Fig. 2A). To assess genetic stability, rMDV-VP2-HA was serially passaged for 20 generations in CEF. Viral genomic DNA was extracted and subjected to PCR identification with primers specific for the HA and VP2 expression cassettes. The results confirmed the consistent amplification of the HA and VP2 gene expression cassettes across all passages (Fig. 2B and C). Furthermore, HA and VP2 protein expression in the serially passaged viruses was validated via IFA and Western blot (Fig. 2D and E). These findings indicate that rMDV-VP2-HA exhibits replication characteristics consistent with the parental virus (rMSΔMeq) and exhibits genetic stability across successive passages.

### rMDV-VP2-HA induces robust humoral and cellular immune responses in immunized chickens

**Fig 3:**
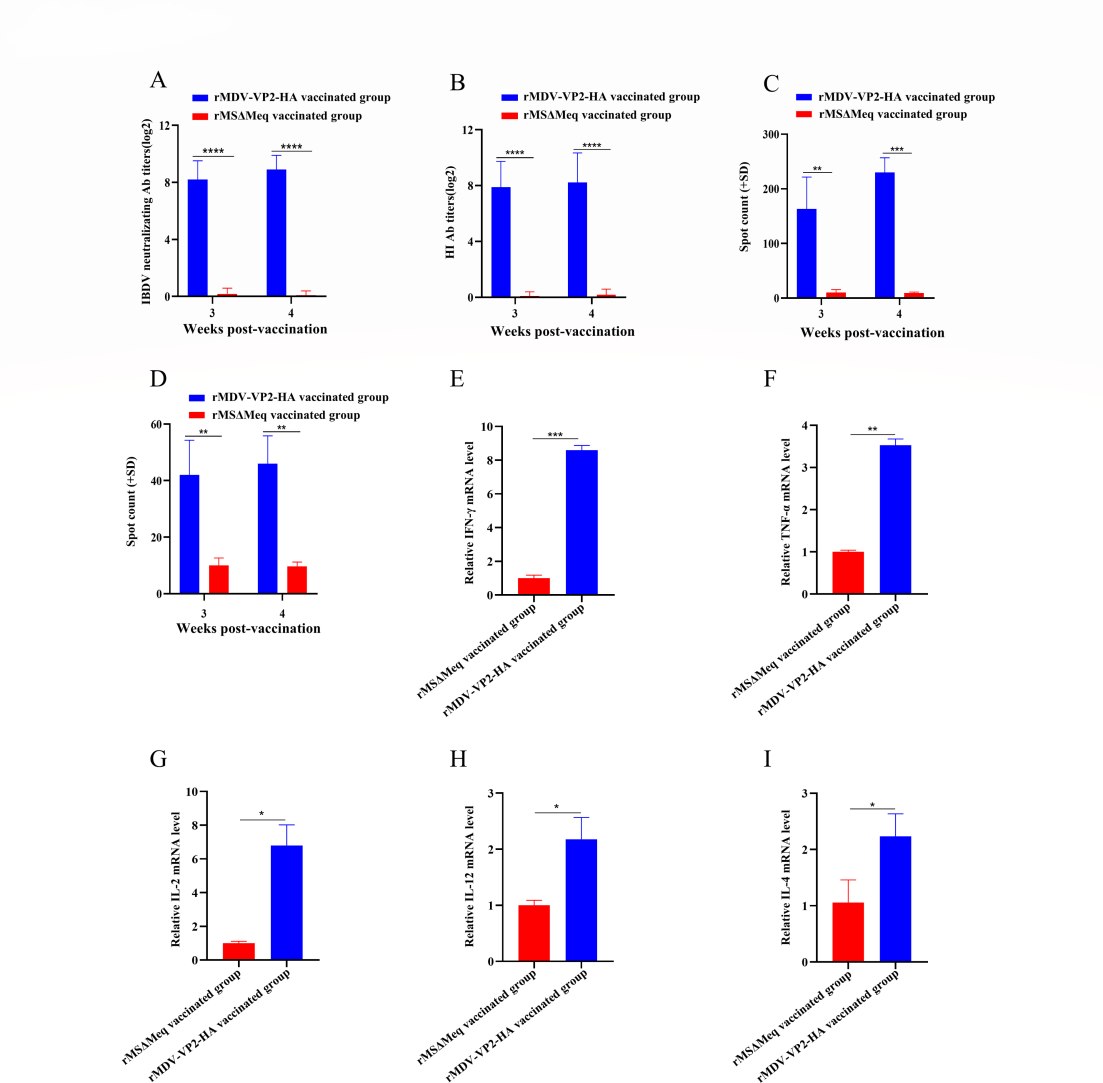
Immune responses against IBDV and H9 subtype AIV in chickens. (**A**) Detection of viral neutralization antibodies against IBDV in chickens at 3 and 4 weeks post-vaccination. (**B**) Detection of HI antibody titer against H9 subtype AIV in chickens at 3 and 4 weeks post-vaccination. (**C and D**) ELISpot assay to measure ch-IFN-γ production by peripheral blood mononuclear cells (PBMCs) of vaccinated chickens with rMDV-VP2-HA. PBMCs of chickens with rMDV-VP2-HA were harvested 3 and 4 weeks post-vaccination. The purified prokaryotic expressed VP2 (5 ng/well) (**C**) and HA (500 ng/well) (**D**) proteins were used to restimulate PBMCs. Spot counts represent the number (mean ± standard deviation) of chIFNγ-secreting cells at each time. Student’s *t*-test was used to compare the differences in means between groups. Symbol (*) denotes differences between the two groups (*P < 0.05, **P < 0.01, and ***P < 0.001). (**E–I**) mRNA expression of cytokines in PBMC at 4 weeks post-vaccination. RT-qPCR assay was used to detect the mRNA expressions of the ch-IFN-γ (**E**), ch-TNF-α (**F**), ch-IL-2 (**G**), ch-IL-12 (**H**), and ch-IL-4 (**I**). Student’s *t*-test was used to compare the differences in means between groups. Symbol (*) denotes differences between the two groups (*P  <  0.05, **P  <  0.01, and ***P <  0.001).

To evaluate the humoral immune response induced by rMDV-VP2-HA, 40 one-day-old SPF chickens were randomly divided into two groups of 20. They were subcutaneously vaccinated with 2,000 PFU of rMDV-VP2-HA or rMSΔMeq and designated as the rMDV-VP2-HA vaccinated group or the rMSΔMeq vaccinated group, respectively. Blood samples were collected at 3 and 4 weeks post-vaccination (w.p.v.) to measure IBDV-neutralizing and HI antibody titers. In the rMDV-VP2-HA vaccinated group, IBDV-neutralizing antibody levels exceeded 8.2 log_2_ at 3 w.p.v. and increased further to an average of 8.9 log_2_ by 4 w.p.v. (Fig. 3A). Similarly, the HI antibody titer of this group increased from 7.9 log_2_ at 3 w.p.v to 8.2 log_2_ at 4 w.p.v (Fig. 3B). In contrast, the rMSΔMeq vaccinated group did not induce any antibody against IBDV and H9 subtype AIV. These results indicate that rMDV-VP2-HA elicits a robust and specific humoral immune response.

To evaluate the cellular immunity of vaccinated chickens, chicken peripheral blood mononuclear cells (PBMCs) were isolated at 3 and 4 w.p.v. and IFN-γ production was measured using the chicken IFN-γ ELISpot assay kit (22). Compared to the rMSΔMeq vaccinated group, PBMCs from chickens vaccinated with rMDV-VP2-HA showed a significant increase in IFN-γ-specific spot numbers after stimulation with both IBDV VP2 protein (5 ng/well) or H9 subtype AIV HA protein (500 ng/well), indicating that rMDV-VP2-HA can activate VP2 and HA-specific cell-mediated immune responses in chickens (Fig. 3C and D). The mRNAs of cytokines in PBMCs at 4 w.p.v. were analyzed. Chickens vaccinated with rMDV-VP2-HA exhibited upregulated expression of key cytokines, including chicken-interferon-γ (ch-IFN-γ), chicken-tumor necrosis factor-alpha (ch-TNF-α), chicken-interleukin-2 (ch-IL-2), chicken-interleukin-12 (ch-IL-12), and chicken-interleukin-4 (ch-IL-4), compared to the rMSΔMeq vaccinated group (Fig. 3E through I). These results indicate that rMDV-VP2-HA can induce an effective cellular immune response.

### rMDV-VP2-HA provides complete protection against the vvIBDV challenge

**Fig 4:**
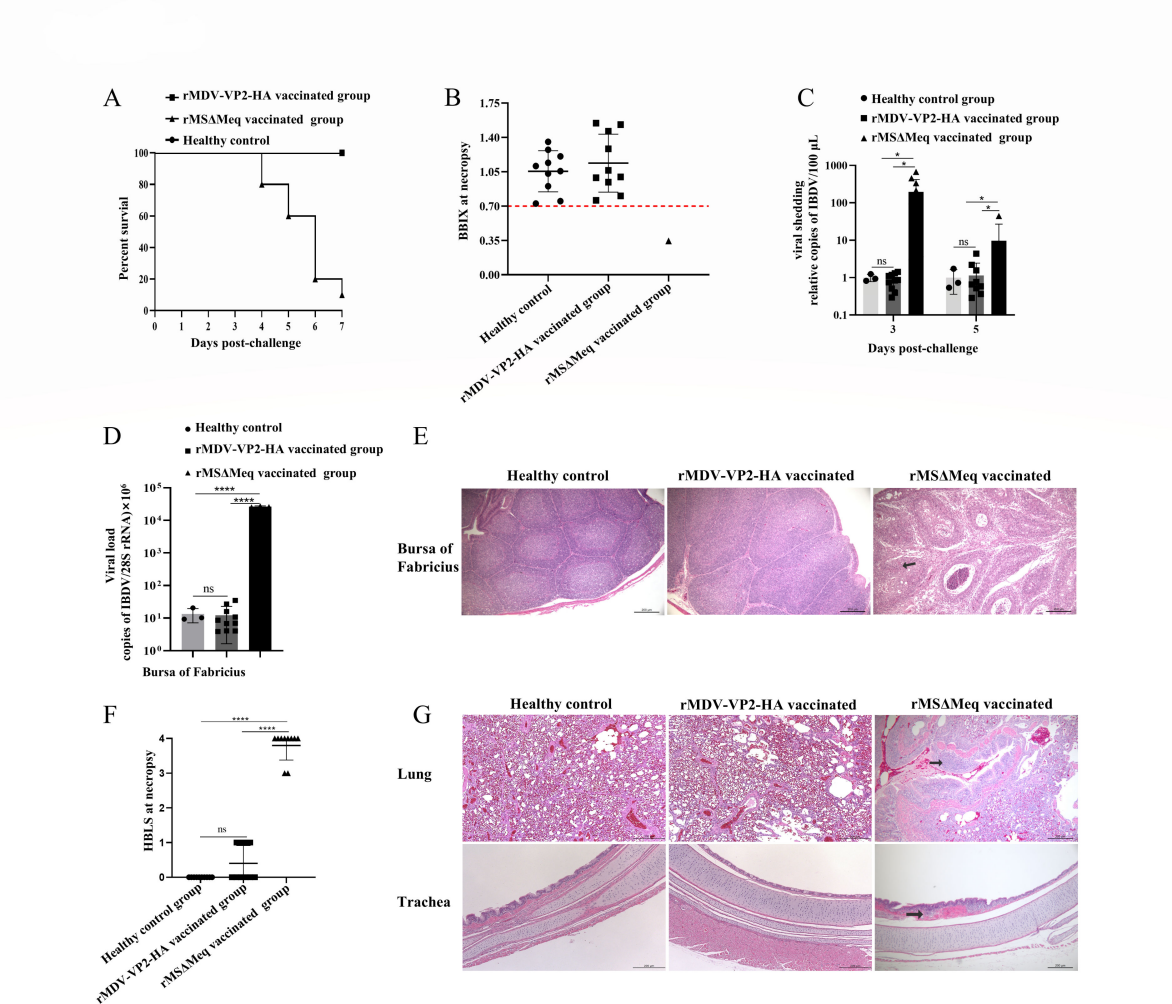
rMDV-VP2-HA provides complete protection against vvIBDV and H9 subtype AIVs challenge. (**A**) Survival rates of chickens challenged with vvIBDV over a 7-day observation period. (**B**) Bursa-to-body weight index (BBIX) values in rMDV-VP2-HA vaccinated group, healthy control group, and rMSΔMeq vaccinated control chickens. (**C**) Relative copies of IBDV in cloacal swab at 3 and 5 days post-challenge (DPC). Cloacal swabs were collected for viral genomic RNA extraction and quantification via RT-qPCR. The data of RT-qPCR are shown as means ± standard deviations (SDs) for triplicates from the representative experiment. The healthy control group was normalized for analysis of viral shedding. The symbol (*) denotes differences between the two groups (*P < 0.05). (**D**) Viral load levels in the bursa post-challenge. Bursa tissues were homogenized 7 DPC for viral genomic RNA extraction and quantification via RT-qPCR. The data of RT-qPCR are shown as means ± SDs for triplicate experiments from the representative experiment. The symbol (*) denotes differences between the two groups (*P < 0.05, **P < 0.01, ***P < 0.001, and ****P < 0.0001). (**E**) Histopathological analysis of bursa of Fabricius samples from chickens that were challenged with vvIBDV at 7 DPC via the respiratory route. The histopathological examination revealed severe atrophy of lymphoid follicles (arrow) in the rMSΔMeq vaccinated group. Scale bar, 200 µm. (**F**) The histopathologic bursal lesion score (HBLS) of chickens that died and survived after challenge. Histopathological lesion score was conducted to quantify the extent of lesions. Data presented are the means ± SDs. (**G**) Histopathological analysis of tracheal and lung samples from chickens that were challenged with H9 subtype AIV 5 DPC via the respiratory route. The histopathological examination revealed inflammatory infiltration (arrow) in the lamina propria and submucosa of the pulmonary bronchioles, as well as in the lamina propria of the tracheal mucosa in the rMSΔMeq vaccinated group. Scale bar, 200 µm.

To evaluate the protective efficacy of the rMDV-VP2-HA against vvIBDV, 10 chickens from both the rMDV-VP2-HA vaccinated group and the rMSΔMeq vaccinated group were challenged with vvIBDV (HLJ-0504 strain) at 28 days post-vaccination (DPV), respectively. The rMDV-VP2-HA vaccinated group exhibited no clinical signs and mortality during the post-challenge observation period (Fig. 4A). In contrast, the rMSΔMeq vaccinated group experienced a 90% mortality rate (9/10 chickens). To evaluate the degree of bursa of Fabricius atrophy, we used the bursa-to-body weight index (BBIX) as a criterion (23). Necropsy findings indicated that the bursa of Fabricius in the rMDV-VP2-HA vaccinated group was normal, with BBIX index values exceeding 0.7, comparable to those in the healthy control group. In the rMSΔMeq vaccinated group, the sole surviving chicken exhibited severe bursa atrophy, with a BBIX index value < 0.7 (Fig. 4B).

To evaluate the efficacy of rMDV-VP2-HA in preventing viral shedding, fecal samples were collected at 3 and 5 days post-challenge (DPC) for viral detection. The copies of IBDV in rMDV-VP2-HA vaccinated chickens were comparable to those in the healthy control group (Fig. 4C). In contrast, the rMSΔMeq vaccinated group showed significantly elevated IBDV copies at 3 and 5 DPC (Fig. 4C). These results indicate that rMDV-VP2-HA can effectively reduce viral shedding following vvIBDV challenge. To evaluate the efficiency of rMDV-VP2-HA in resisting virus replication, the bursa of Fabricius were collected from chickens 7 DPC to assess the viral load. In the rMSΔMeq vaccinated group, the viral load of IBDV was 2.7 × 10⁴ copies per 10⁶ cells in the bursa of Fabricius. In contrast, the viral loads in the rMDV-VP2-HA vaccinated group were comparable to those in the healthy control group (negative for detectable IBDV). This indicated that rMDV-VP2-HA effectively blocked viral replication in the bursa of Fabricius (Fig. 4D).

Histopathological examination of the bursa of Fabricius confirmed normal histology in the rMDV-VP2-HA vaccinated group and healthy control groups, without observable histopathological changes (Fig. 4E), and the rMDV-VP2-HA vaccinated group demonstrated no histopathologic bursal lesions (HBLS = 0.4) (Fig. 4F), whereas the rMSΔMeq vaccinated group showed severe lymphoid follicle atrophy and lymphocyte depletion (HBLS = 3.8) (Fig. 4E and F). These findings demonstrate that rMDV-VP2-HA can provide complete immune protection against vvIBDV challenge.

### rMDV-VP2-HA provides complete protection against H9 subtype AIV challenge

To evaluate the protective efficacy of the rMDV-VP2-HA against H9 subtype AIV, another 10 chickens from both the rMDV-VP2-HA vaccinated group and the rMSΔMeq vaccinated group were challenged with the H9 subtype AIV (A/CK/GX/S11583/2019 strain) at 28 DPV, respectively. The rMDV-VP2-HA vaccinated group exhibited no H9 subtype viral shedding in oropharyngeal or cloacal swabs at 3 or 5 DPC (Table 1). In contrast, the rMSΔMeq vaccinated group demonstrated a 100% incidence of viral shedding at both time points. Further histopathological examination revealed no observable histopathological damage to the lungs or trachea in the rMDV-VP2-HA vaccinated group. In contrast, the rMSΔMeq vaccinated group exhibited inflammatory cell infiltration in the lungs and trachea tissues (Fig. 4G). These findings suggest that rMDV-VP2-HA provides complete protection against H9 subtype AIV, including associated respiratory tract pathology.

**TABLE 1:** Recombinant virus (rMDV-VP2-HA) protection against H9 subtype AIV

Group	Challenge strain	Virus shedding of challenged chickens				Protected count/total count	Protection rate
		Oropharynx 3 DPC	Cloaca 3 DPC	Oropharynx 5 DPC	Cloaca 5 DPC		
Healthy control group	–^*a*^	0/10	0/10	0/10	0/10	10/10	100%
rMDV-VP2-HA vaccinated group	CK/GX/S11583/2019	0/10	0/10	0/10	0/10	10/10	100%
rMSΔMeq vaccinated group		10/10	6/10	10/10	10/10	0/10	0%

^*a*^ –, chickens without CK/GX/S11583/2019 challenge.

### rMDV-VP2-HA provides complete protection against vvMDV challenge

**Fig 5:**
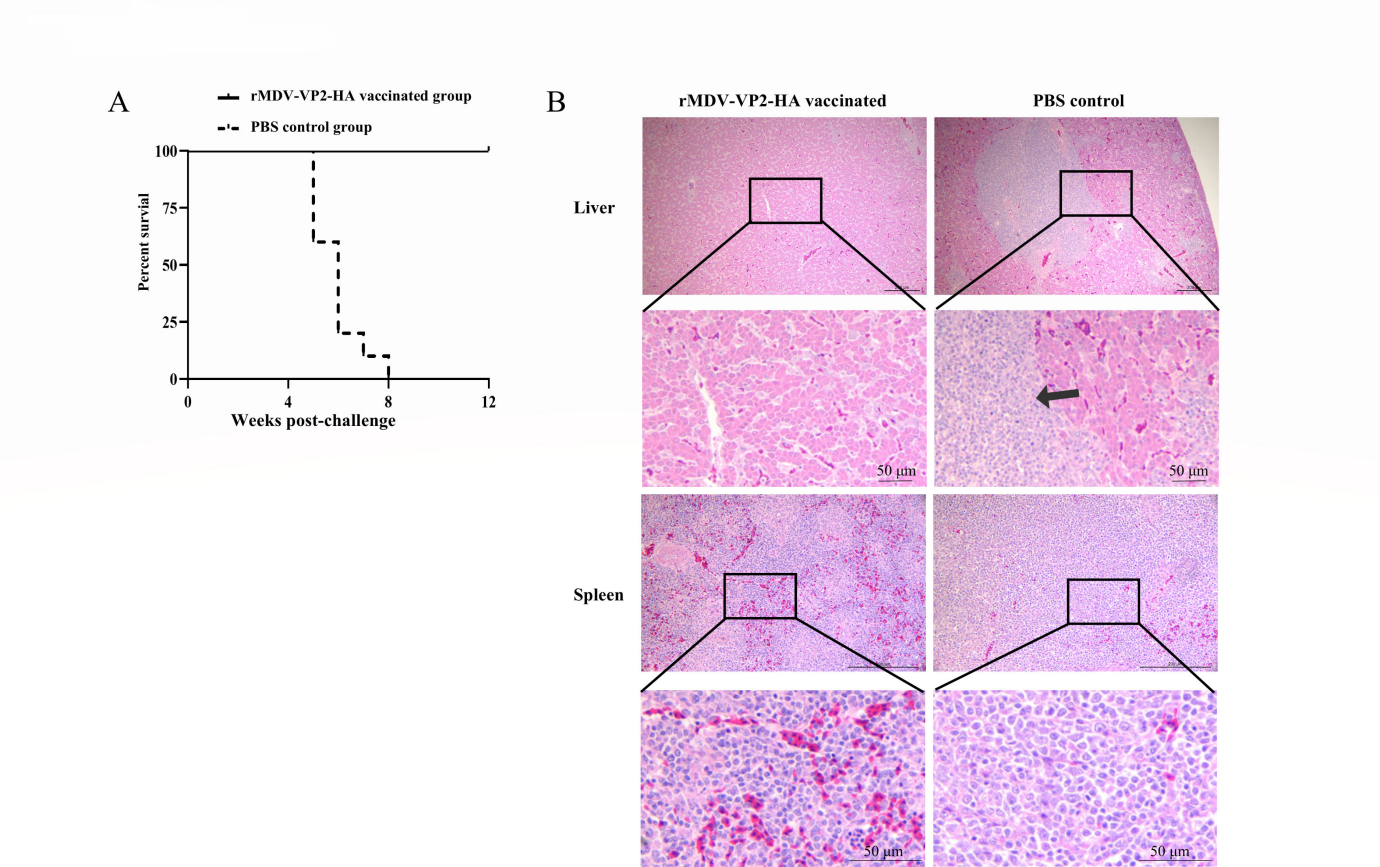
rMDV-VP2-HA provides complete protection against the vvMDV challenge. (**A**) Survival rates of chickens challenged with vvMDV over a 12-week observation period. All chickens survived without any clinical symptoms, and no tumor formation was observed in any organs. (**B**) Histopathological examination in chickens of the rMDV-VP2-HA vaccinated group and the PBS control group. Hematoxylin and eosin (H&E) staining was performed on spleen and liver from chickens in the PBS control group (collected immediately after death post-challenge) and the rMDV-VP2-HA vaccinated group (euthanized at 12 weeks post-challenge). Histopathological examination revealed focal infiltration of neoplastic cells within the parenchyma in the PBS control group (arrows and localized magnification). Scale bar, 200 µm.

To evaluate the protective efficacy of the rMDV-VP2-HA against vvMDV, 20 one-day-old SPF chickens were randomly divided into two groups of 10. One group was subcutaneously vaccinated with 2,000 PFU of rMDV-VP2-HA as rMDV-VP2-HA vaccinated group, and the other received a subcutaneous injection of PBS as the PBS control group. The rMDV-VP2-HA vaccinated group and the PBS control group were challenged with the vvMDV (Md5 strain) at 7 DPV and observed for 12 weeks. All chickens in the PBS control group developed MD lesions and exhibited marked liver and spleen pathology, resulting in 100% mortality (Fig. 5A). In contrast, the rMDV-VP2-HA vaccinated group exhibited no clinical signs of infection and no MD lesion, providing a protective index (PI) of 100 against Md5 (Table 2). Histopathological examination demonstrated that the livers of the PBS control group exhibited tumor cell infiltration within the parenchyma, while the spleens showed pronounced tumor cell proliferation and infiltration (Fig. 5B). These results demonstrate that the rMDV-VP2-HA can provide complete protection against vvMDV.

**TABLE 2:** Protection of rMDV-VP2-HA against vvMDV Md5

Group	Vaccine strain	MD mortality	MD lesion	PI^*a*^
rMDV-VP2-HA vaccinated group	rMDV-VP2-HA	0/10 (0)	0/10	100
PBS control group	–^*b*^	10/10 (100)	10/10 (100)	0

^*a*^ PI, protective index.

^*b*^ –, chickens without vaccination.

## DISCUSSION

MDV vectors, characterized by their large genome and multiple insertion regions, have been extensively studied due to their ability to induce lifelong immunity post-infection without interference from maternal antibodies (24, 25). Among the MDV serotypes, serotype 3 MDV vector, exemplified by HVT, has been extensively employed as a vector (26–28), particularly in recombinant virus. Examples include HVT-ND-IBD, co-expressing the Newcastle disease virus (NDV) F protein and IBDV VP2 protein (29), and HVT-IBD-AI, co-expressing the IBDV VP2 protein and H5 subtype AIV HA protein (30). Although these recombinant viruses confer robust protection against inserted foreign pathogens, their protection against MDV remains suboptimal. Achieving adequate resistance often necessitates co-immunization with MDV-1 vaccines such as CVI988 or 814 strains. Consequently, attenuated MDV-1, providing strong protection against vvMDV strains, is regarded as an ideal viral vector for recombinant vaccine development (31). In this study, we utilized a previously developed attenuated MDV-1 vaccine strain (rMSΔMeq) as a vector to construct the recombinant virus rMDV-VP2-HA. This recombinant virus has been proven to effectively protect against currently circulating MDV strains. The results indicated that rMDV-VP2-HA induced a robust humoral immune response, characterized by high levels of IBDV-neutralizing antibodies and HI antibodies, alongside specific cellular immunity in response to the stimulation of VP2 and HA. Challenge experiments showed that rMDV-VP2-HA conferred complete protection against IBDV, H9 subtype AIV, and vvMDV challenges. These findings underscore the potential of rMDV-VP2-HA as a promising multivalent vaccine candidate capable of simultaneously controlling IBD, H9 subtype AI, and MD.

The genetic stability of the construct is essential for the development of MDV vaccines that incorporate multiple gene insertions. Key factors influencing stability include the sequence of the expression cassette, comprising the promoter and inserted sequences, as well as the specific genome insertion region within the MDV genome. Previous studies have demonstrated that the US2 and UL41 regions of MDV are favorable regions for the insertion of foreign genes, demonstrating robust expression levels (31). In this study, the HA gene was inserted into the UL41 region and the VP2 gene into the US2 region. Among the promoters tested, the SV40 promoter for HA and the CMV promoter for VP2 yielded optimal protective efficacy. *In vitro*, replication dynamics studies showed that the growth curve of the recombinant virus was consistent with that of the parental virus. Importantly, after 20 consecutive *in vitro* passages, the HA and VP2 genes remained stably inherited, demonstrating the high genetic stability of the recombinant virus. These findings provide the first demonstration that MDV serotype 1 can concurrently and stably express multiple foreign genes. This highlights its potential as a robust viral vector for developing multivalent vaccines.

The speed and magnitude of antibody production are critical indicators of vaccine efficacy. Early vaccination provides rapid protection, which is crucial for preventing H9 AIV infection. Unlike other H9 AIV vaccines such as rL-H9 and rFPV282-12LSH9A, which are typically administered to chicks aged 1–4 weeks (32–34), rMDV-VP2-HA can be given at 1 day of age. Notably, it induces detectable antibodies within 2 weeks post-vaccination. Moreover, the rMDV-VP2-HA developed in this study demonstrates a consistent increase in HI antibody titers, achieving and maintaining levels of approximately 8 log_2_ at 3–4 weeks post-immunization. These titers are significantly higher than those induced by existing recombinant HA protein vector vaccines, such as HVT-HA, which typically elicit HI antibody titers of 3–6 log_2_ (17, 35, 36). This elevated antibody response ensures enhanced protection against H9 subtype AIV challenges. In summary, the rMDV-VP2-HA developed in this study provides rapid and effective protection against H9 subtype AIV, ensuring critical protection during the early vulnerable stages of chickens. The sustained high HI antibody titers ensure long-lasting immunity, representing a major advancement in poultry disease prevention and control.

As the sole capsid protein and main protective antigen of IBDV, VP2 has been widely shown to induce effective protective immunity in various vector systems. However, previous studies using the rMSΔMeq vaccine strain as a vector to express the IBDV VP2 gene reported limited efficacy. Specifically, one such recombinant virus, when administered to 1-day-old chickens, elicited only a neutralizing antibody titer of 5 log_2_ and provided 50% survival protection against vvIBDV challenge, without protection against bursal atrophy (37). In contrast, our recombinant virus rMDV-VP2-HA, despite carrying two foreign genes, induced significantly higher levels of neutralizing antibody than the recombinant virus rLMSΔMeq-VP2 when immunized chickens were tested 28 days later (8.9 log_2_ >5 log_2_). Furthermore, rMDV-VP2-HA conferred complete protection against vvIBDV, effectively preventing both mortality and bursal atrophy. This enhanced protective efficacy may stem from the distinct promoters driving the expression of the VP2 gene. Although the previous recombinant virus utilized the chicken β-actin promoter, we employed the CMV promoter to drive VP2 gene expression. Further research is warranted to construct recombinant virus expressing VP2 under different promoters to elucidate the precise role of promoter selection in optimizing protective immunity.

The Th1 and Th2 immune responses are key to the long-lasting protective immunity and protective efficacy elicited by vaccines. The Th2-type immune response, driven by IL-4, is implicated in the stimulation of the humoral response. In our study, the rMDV-VP2-HA vaccinated group showed elevated IL-4 transcription, confirming the activation of the Th2-type immune response. The Th1-type immune response, driven by IFN-γ, is essential for eliminating intracellular pathogens. IFN-γ production is typically used as a marker for Th1 cells activity (38). Studies have shown that the recombinant rHVT-H9 vaccine elicits a strong cellular immune response, as evidenced by the detection of IFN-γ^+^/CD4^+^ and IFN-γ/CD8^+^ T cells at 5 weeks post-vaccination (17). Similarly, our findings indicate that chickens vaccinated with the rMDV-VP2-HA exhibited significantly higher IFN-γ levels in PBMCs following stimulation with IBDV VP2 and H9 subtype AIV HA protein stimulation at 3 and 4 weeks post-immunization. Additionally, transcriptional upregulation of multiple Th1-type cytokines was observed in the vaccinated group compared to the rMSΔMeq vaccinated control group. These results indicate that rMDV-VP2-HA vaccination elicits a strong cellular immune response, contributing to broad protection against viral infections. Collectively, rMDV-VP2-HA simultaneously elicits both Th1 and Th2 immune responses.

In conclusion, this study presents the first construction of a recombinant virus, rMDV-VP2-HA, using the MDV-1 vaccine strain (rMSΔMeq) as a vector to co-express the IBDV VP2 gene and the H9 subtype AIV HA gene. This recombinant virus exhibited excellent genetic stability and elicited robust humoral and cellular immune responses in immunized chickens. Importantly, rMDV-VP2-HA provided complete protection against challenges with H9 subtype AIV, IBDV, and MDV challenges, establishing its potential as a promising multivalent vaccine candidate for the simultaneous control of IBD, H9 subtype AI, and MD.

## MATERIALS AND METHODS

### Viruses and cells

The MDV-1 rMSΔMeq vaccine strain (39) was used as the parental virus for producing recombinant MDV. The vvIBDV HLJ-0504 strain (40) and the H9 subtype AIV A/CK/GX/S11583/2019 strain were used as challenge viruses. MDV was propagated in CEFs derived from 8-day-old SPF chicken embryos.

### Construction of fosmids with HA and VP2 gene insertion

To obtain a recombinant fosmid, the VP2 gene from the vvIBDV (HLJ-0504 strain) was cloned into the pEGFP-N1 vector, and the EGFP ORF was replaced to form the VP2-expressing cassette under the control of the CMV promoter. Similarly, the HA gene from the H9 subtype (A/CK/GX/S11583/2019) strain was cloned into pcDNA3.1, under the control of the SV40 promoter. Five fosmid clones covering the complete genome of the MDV-1 rMSΔMeq vaccine strain, designated pMDV1-pMDV5, were maintained in our laboratory (20). Recombinant pMDV3-UL41/HA fosmids were generated using a two-step Red/ET-mediated recombination. In step 1, the antibiotic selection cassette (rpsL-neo) was amplified using PCR with specific primers containing 50 bp homology arms of the UL41 insertion region (Table 3). PCR products and pMDV3 fosmid were electroporated into DH10B competent cells. In step 2, the HA-expressing cassette under the control of the SV40 promoter was PCR-amplified using the primers listed in Table 3 and electroporated into rpsL-neo-positive competent cells to replace rpsL-neo. Only fosmids with the rpsL-neo marker were replaced with the HA-expressing cassette, replicated, and selected for DH10B. All the recombinant fosmids were sequenced. The method for obtaining the recombinant plasmid pMDV4-US2/VP2 was similar to the aforementioned procedure.

**TABLE 3:** Primers

Primer	Sequence（5′−3′)
UL41-rpsl F	CACATCCCATTAATATCAAATCTGTGTCCGAAGACAATACATATGCGACGGCCTGGTGATGATGGCGGGATCG
UL41-rpsl R	CACATCCCATTAATATCAAATCTGTGTCCGAAGACAATACATATGCGACTCAGAAGAACTCGTCAAGAAGGCG
UL41-SV40 F	CACATCCCATTAATATCAAATCTGTGTCCGAAGACAATACATATGCGACGTGTGTCAGTTAGGGTGTGG
UL41-SV40 R	GACTACATGCACGATTGTTTGATGAATGTGGAAGTACGGATGTTGGAGAGTAAGATACATTGATGAGTTT
US2-rpsl F	TCTGTCGAATAACAGCTAATGACTACCGGGTGTGTCAGTTAGGGTGTGGGGCCTGGTGATGATGGCGGGATCG
US2-rpsl R	CTGTCGAATAACAGCTAATGACTACCGGGTGTGTCAGTTAGGGTGTGGTCAGAAGAACTCGTCAAGAAGGCG
US2-CMV F	ATCTAATTGGTAGCAAGTAGGTCTGTCGAATAACAGCTAATGACTACCGGCGTTACATAACTTACGGTAA
US2-CMV R	TGGGTGTGCCCATAATCGTCAGAGCTGCAGACCTATTCCGTTTTGCCAAATTTGAGTAGTTACATAGAAT
HA-F	TATTCCAGAAGTAGTGAGGA
HA-R	GGCGAAGAACTCCAGCATGA
VP2-F	ATGACAAACCTGCAAGATCA
VP2-R	TTACCTTAGGGCCCGAATTA
IFN-γ F	ACTGACAAGTCAAAGCCGCACA
IFN-γ R	TCGTTCATCGGGAGCTTGGC
TNF-α F	GGGAATGAACCCTCCGCAGT
TNF-α R	CCACCACACGACAGCCAAGT
IL-2 F	AACTGGGACACTGCCATGAT
IL-2 R	TCCTGGGTCTCAGTTGGTGT
IL-12 F	ACGTCACCAACAGTCAGAGC
IL-12 R	GGTCTTCGTAGATCCCCTGC
IL-4 F	AACATGCGTCAGCTCCTGAA
IL-4 R	AGGTACGTAGGTCTGCTAGGA
β-actin F	CACAGATCATGTTTGAGACCTT
β-actin R	CATCACAATACCAGTGGTACG

### Rescue of recombinant virus from overlapping fosmid DNAs

To rescue the recombinant virus, five fosmids containing recombinant plasmids (pMDV1, pMDV2, pMDV3-UL41/HA, pMDV4-US2/VP2, and pMDV5) were purified with NotI-HF (Neb and R3189L) and co-transfected into primary CEF to rescue the recombinant virus by calcium phosphate transfection (Invitrogen, K278001) (41). After 6 h of transfection, the cell supernatant was replaced with DMEM supplemented with 2% fetal bovine serum (FBS) and 1% streptomycin and penicillin (SP) for continued incubation.

### Confirmation of the HA and VP2 gene expression

To identify the HA and VP2 gene expression, the recombinant virus and the parental virus (rMSΔMeq) were inoculated into CEF cells cultured in six-well plates at a multiplicity of infection (MOI) of 100 plaque-forming units (PFU). After 120 h of incubation, the cells were collected for analysis by IFA. The IFA protocol involved an initial incubation of the cells with polyclonal anti-HA antibodies obtained from chicken for 1 h at 37°C. This was followed by a 1-h incubation with monoclonal anti-VP2 antibodies obtained from mouse at 37°C, specific for the viral VP2 protein. Subsequently, the cells were incubated with a combination of FITC-conjugated anti-chicken IgG (Sigma-Aldrich, F4137) and TRITC-conjugated anti-mouse IgG (Abcam, ab6786) for 1 h at 37°C, enabling the detection of both chicken and mouse immunoglobulins that had bound to their respective antigens.

Western blot analysis was utilized to assess the expression of HA and VP2 proteins. CEFs were cultured in six-well plates and infected with either the recombinant virus or the parental virus (rMSΔMeq) at an MOI of 100 PFU, or left uninfected as a control. After a 4-day incubation, the cells were lysed and subjected to SDS-PAGE. Proteins were transferred to a nitrocellulose membrane and blocked with 5% non-fat milk for 30 min at 4°C. The membranes were then probed with HA polyclonal, VP2 monoclonal, and glycoprotein I (gI) monoclonal antibodies for 1.5 h at room temperature. After multiple washes with PBST, the membranes were incubated with IRDye 800CW donkey anti-chicken antibody (LI-COR, 925-32218) and IRDye 800CW Goat anti-Mouse IgG (LI-COR, 926-32210) for 1 h. The protein bands were visualized using an infrared imaging system (LI-COR, Odyssey CLX) after the final wash.

### Growth properties and stability of the rescued viruses

To assess the replication competency of the recombinant virus, CEFs grown in six-well plates were respectively inoculated with 100 PFU of the recombinant virus and parental virus (rMSΔMeq). At 24, 48, 72, 96, 120, and 144 h post-inoculation, the cells were harvested and passaged by serial dilution onto fresh CEFs. Six days after passage, plaque formation was quantified for each dilution to determine the viral growth curve.

To evaluate the genetic stability of the recombinant virus, the virus was subjected to 20 serial passages in CEFs. The presence of the inserted VP2 and HA genes was ascertained by PCR amplification and subsequent sequencing. Expression of VP2 and HA genes was further validated by Western blot and IFA, as described above.

### Examination of humoral immunity

60 one-day-old SPF chickens were randomly divided into three groups of 20 birds each. The rMDV-VP2-HA vaccinated group received the rMDV-VP2-HA, at a dosage of 2,000 PFU per chick. The rMSΔMeq vaccinated group was vaccinated with the parental virus (rMSΔMeq) at the same dosage. The unvaccinated group served as the healthy control group. Serum samples were collected from the chickens weekly for subsequent analysis.

To perform the IBDV neutralization test, heat-inactivated serum samples were prepared and serially diluted twofold, creating triplicate sets. Each diluted serum sample was mixed with an equal volume of cell-adapted HLJ-0504 strain, adjusted to a titer of 200 TCID_50_. The mixtures were incubated for 60 min at 37°C to allow antibody-virus interactions. Following incubation, the serum-virus mixtures were introduced onto monolayers of CEFs in culture and incubated for 3 days to assess cytopathic effects (CPEs). The viral neutralization titer was determined as the log_2_ of the reciprocal of the highest serum dilution that resulted in the absence of detectable CPE, indicating effective virus neutralization by the immune serum.

The HI test can partially reflect the protective efficacy. The brief steps are as follows. Add 25 µL of phosphate-buffered saline (PBS) to each of wells 1–11 in the hemagglutination plate, and 50 µL to well 12. Pipette 25 µL of serum into the first well of each row and mix gently by pipetting six to eight times. Then, successively pipette 25 µL of the liquid from one well to the next. Well 11 is set as the antigen control, and well 12 is set as the red blood cell control. Add 25 µL of antigen (four hemagglutination units) successively from right to left to the first 11 wells, and gently shake the plate at room temperature for 30 min. Next, pipette 25 µL of 1% chicken red blood cell suspension into each well of the 96-well plate and mix gently at room temperature for 25 min. Finally, calculate the antibody titer of the serum as the reciprocal of the highest dilution that produced a disappearance of red blood cell agglutination.

### PBMC isolation

PBMCs were isolated using Histopaque-1119 (Sigma, 11191). Briefly, add the anticoagulant to the upper layer of Histopaque-1119, centrifuge at 1,000 × *g* for 15 min to isolate the PBMC layer, and then wash the PBMC cells with 0.5% FBS PBS. After washing the PBMCs, 200 µL of PBMCs at a concentration of 1 × 10^7^/mL was directly used for ELISpot experiments to assess cytokine production. Another 200 µL of PBMCs was mixed with 1 mL of TRIzol reagent for subsequent RNA extraction and cytokine detection. Remaining PBMCs were centrifuged, and the cell pellet was resuspended in a freezing medium (90% FBS and 10% dimethyl sulfoxide) at a concentration of 1 × 10^6^ cells/mL. These cells were then frozen in liquid nitrogen for long-term storage and used for subsequent experiments as needed.

### Examination of cell-mediated immunity

An ELISpot assay was performed to measure ch-IFN-γ production using Ch-IFN-γ ElispotPLUS kit (HRP) (Mabtech, 3125-2H) following the manufacturer’s protocol (22). The 96-well plate was pre-activated with 35% ethanol and coated with captured antigens, followed by overnight incubation. Afterward, the plate was washed and then incubated with RPMI-1640 medium supplemented with 10% FBS. PBMCs were seeded at 5 × 10^5^ cells/well, with triplicates in RPMI-1640. The purified IBDV VP2 protein (5 ng/well) and purified HA protein (500 ng/well) expressed in prokaryotic systems were used to stimulate the PBMCs. The plate was incubated at 37°C with 5% CO_2_ for 48 h. Ch-IFN-γ was detected by incubation with biotinylated mouse-anti-Ch-IFN-γ (MT&C10-biotin) and Streptavidin-HRP (1:1,000). Color development was achieved by adding 100  µL TMB (Mabtech, 3651-10) in the dark until distinct spots appeared. Spots were quantified and analyzed using an ELISpot reader (AID, Germany).

Real-time PCR assay was used to detect cytokine mRNA expressions. Total mRNA from PBMCs was extracted using RNAiso Plus (TaKaRa, 9109) and immediately transcribed to cDNA using HiScript III qRT Supermix (Vazyme, R223-01). Real-time RT-PCR assays were performed to quantify the mRNA level of ch-IFN-γ, ch-TNF-α, ch-IL-2, ch-IL-12, and ch-IL-4 using the resultant cDNA, SYBR qPCR Mix (Toyobo, TYB-QPS-201) and specific primers (Table 3). Chicken-β-actin (ch-β-actin) was used as a housekeeping gene.

### Protection against vvIBDV challenge

To assess the protective efficacy of the recombinant virus against vvIBDV, 30 one-day-old SPF chickens were randomly selected. Ten chickens were vaccinated with 2,000 PFU of the recombinant virus by subcutaneous injection, designated as the rMDV-VP2-HA vaccinated group. Ten chickens were vaccinated with 2,000 PFU of the parental virus by subcutaneous injection, designated as the rMSΔMeq vaccinated group. The remaining 10 unvaccinated chickens served as a healthy control group. At 28 DPV, all groups except the healthy control group were challenged with a viral dose of 10 CID_50_/bird of vvIBDV, with a challenge volume of 100 µL, via nasal droplet and ocular instillation. After challenging, the chickens were monitored daily for a week to record mortality and morbidity rates across all groups. At 7 DPC, the chickens were humanely euthanized, and the bursa of Fabricius were harvested for further analysis. The BBIX, a critical metric for evaluating bursal immune competence in poultry, was calculated as: BBIX = (vaccinated group bursa weight/body weight [gram])/(healthy control group bursa weight/body weight [gram]) × 1,000 (23). BBIX values were compared between the test group and the healthy control group. A BBIX value < 0.7 indicated bursal atrophy (42), while a value > 0.7 suggested normal bursa morphology.

Bursa of Fabricius (7 DPC) and swabs (3 and 5 DPC) were processed for RNA extraction using RNAiso Plus (TaKaRa, 9109), followed by reverse transcription (RT) into cDNA using HisScript III qRT Supermix (Vazyme, R223-01). In the reaction, 12 µL of extracted RNA and 4 µL of 4 × gDNA wiper Mix were heated at 42°C for 2 min and then 5 × HiScript III qRT Supermix 4 µL was added to this reaction mixture. The RT reaction mixture was incubated in this sequence: 50°C for 15 min and 85°C for 5 s. RT-PCR was performed using the target gene forward primer VP5-F (5′-GAGCCTTCTGATGCCAACAAC-3′), the reverse primer VP5-R (5′-CAAATTGTAGGTCGAGGTCTCTGA-3′), and the IBDV probe (5′FAM-CGGCGTCCATTCCGGACGAC-3′BHQ-1). The housekeeping gene forward primer 28S-F (5′-GGCGAAGCCAGAGGAAACT-3′), the reverse primer 28S-R (5′-GACGACCGATTTGCACGTC-3′), and the 28S probe (5′FAM-AGGACCGCTACGGACCTCCACCA-3′TAMRA) (Premix Ex Taq) (Probe qPCR)^*2^ (Takara, RR390A). The viral gene copy numbers in tissues were calculated and expressed as copies of IBDV RNA/28S rRNA ×10^6^ cells, in accordance with a previous study (43, 44). The viral gene copy numbers detected in cloacal swabs were quantified as copies of IBDV RNA per 100 µL and the data from the healthy control group were normalized for analysis of viral shedding (45).

Bursa of Fabricius harvested from vvIBDV-challenged chickens was fixed in 10% phosphate-buffered formalin for subsequent histological analysis. The tissue samples were processed for paraffin embedding, and 5 µm sections were obtained and stained with hematoxylin and eosin (H&E) to assess microscopic structural changes and identify pathological features.

### Protection against H9 subtype AIV challenge

To assess the protective efficacy of the recombinant virus against H9 subtype AIV, 30 one-day-old SPF chickens were randomly selected. Ten chickens were vaccinated with 2,000 PFU of the recombinant virus by subcutaneous injection, designated as the rMDV-VP2-HA vaccinated group. 10 chickens were vaccinated with 2000 PFU of the parental virus by subcutaneous injection, designated as the rMSΔMeq vaccinated control group. The remaining 10 unvaccinated chickens served as a healthy control group. At 28 DPV, all groups except the healthy control group were challenged with a viral dose of 10 CID_50_/bird of the H9 subtype AIV (A/CK/GX/S11583/2019), with a challenge volume of 100 µL, through nasal instillation. At 3 and 5 DPC, swabs were collected from the pharynx and cloaca of each chicken and inoculated into the allantoic cavities of 10-day-old SPF embryonated eggs. The eggs were candled daily and incubated for 48 h. The HA titer was determined, and an HA titer of ≥1:16 (micro method) was considered positive for viral isolation, indicating active viral shedding. Tracheal and lung tissues from chickens challenged with A/CK/GX/S11583/2019 at 5 DPC were fixed in 10% phosphate-buffered formalin for subsequent histological analysis.

### Protection against vvMDV challenge

To assess the protective efficacy of the recombinant virus against vvMDV, 20 one-day-old SPF chickens were randomly selected. Ten chickens were vaccinated with 2,000 PFU of the recombinant virus by subcutaneous injection, designated as the rMDV-VP2-HA vaccinated group. The remaining 10 unvaccinated chickens served as the unvaccinated challenge control group. At 7 DPV, both groups were challenged with 1,000 PFU of the vvMDV strain Md5 by intraperitoneal injection. The chickens were examined for mortality, MD lesion, PI for 12 weeks after the challenge. The dead birds were dissected on the day of death, and tissues with MD lesions were fixed in 10% phosphate-buffered formalin for subsequent histological analysis. Meanwhile, the rMDV-VP2-HA vaccinated group was humanely sacrificed at the end of the 12-week observation period for further analysis.

### Statistical analysis

All data were analyzed using GraphPad Prism 8.0. Results are presented as the mean ± standard deviation (*M* ± SD). Comparisons between groups were performed using an independent samples *t*-test, with statistical significance set at a *P* value of <0.05.

## Data Availability

All data are fully available without restriction.
